# Resolution of inflammation: therapeutic potential of pro-resolving lipids in type 2 diabetes mellitus and associated renal complications

**DOI:** 10.3389/fimmu.2012.00318

**Published:** 2012-10-18

**Authors:** Emma Börgeson, Catherine Godson

**Affiliations:** UCD Diabetes Research Centre, UCD Conway Institute, School of Medicine and Medical Sciences, University College DublinDublin, Ireland

**Keywords:** inflammation, resolution, lipxoins, resolvins, protectins, renal inflammation

## Abstract

The role of inflammation in the pathogenesis of type 2 diabetes mellitus (T2DM) and its associated complications is increasingly recognized. The resolution of inflammation is actively regulated by endogenously produced lipid mediators such as lipoxins, resolvins, protectins, and maresins. Here we review the potential role of these lipid mediators in diabetes-associated pathologies, specifically focusing on adipose inflammation and diabetic kidney disease, i.e., diabetic nephropathy (DN). DN is one of the major complications of T2DM and we propose that pro-resolving lipid mediators may have therapeutic potential in this context. Adipose inflammation is also an important component of T2DM-associated insulin resistance and altered adipokine secretion. Promoting the resolution of adipose inflammation would therefore likely be a beneficial therapeutic approach in T2DM.

## INFLAMMATION AND COUNTER-REGULATORY LIPID MEDIATORS

The inflammatory response is necessary for effective host defense, although it must eventually dissipate to ensure tissue homeostasis and avoid pathologic conditions such as abscess formation, scarring, fibrosis, and eventual organ failure (Lawrence and Gilroy, 2007). Indeed, compromised resolution has been proposed as an underlying mechanism in many prevalent chronic diseases such as arthritis, diabetes, and atherosclerosis (Serhan et al., 2008; Maderna and Godson, 2009). It is now recognized that the resolution of inflammation is a dynamically regulated process orchestrated by mediators that play important counter-regulatory roles including cytokines, chemokines, and lipid mediators such as the lipoxins (LXs), resolvins, and protectins (Serhan, 2009). These mediators reduce vascular permeability and inhibit polymorphonuclear cell (PMN) recruitment, while promoting recruitment of monocytes and stimulating efferocytosis (Serhan et al., 2008). It has also been proposed that pro-resolving lipids stimulate lymphatic drainage of leukocytes (Arita et al., 2005b). Interestingly, the signaling pathways initially inducing prostaglandin (PG)E_2_ and PGD_2_ formation and thus the onset of inflammation, may actively switch the production of lipid mediators from pro-inflammatory to pro-resolving by inducing 5-lipoxygenases (LO) necessary for production of LXs, protectins, and resolvins (Serhan and Savill, 2005). In this way physiological inflammation programs its own resolution and promotes tissue homeostasis (Levy et al., 2001).

## LIPOXINS

The LXs are produced endogenously at sites of inflammation as counter-regulatory lipid mediators with anti-inflammatory, pro-resolving, and anti-fibrotic bioactions (Serhan et al., 2008; Maderna and Godson, 2009). LXs are typically generated by transcellular metabolism between neutrophils, platelets, and resident tissue cells, such as epithelial cells (Lefer et al., 1988; Serhan, 2007), through the sequential action of 5-LO and either 12-LO or 15-LO (Serhan, 2005; Parkinson, 2006). LXs limit leukocyte chemotaxis (Lee et al., 1989) and activation of neutrophils and eosinophils (Bandeira-Melo et al., 2000), while stimulating Mφ efferocytosis of apoptotic cells (Godson et al., 2000; Mitchell et al., 2002; Reville et al., 2006). Lipoxin A_4_ (LXA_4_) and its positional isomer lipoxin B_4_ (LXB_4_) are the principal LX species found in mammals. Although the LXB_4_ receptor remains to be identified, the LXA_4_ receptor FPR2/ALX is expressed on cells of diverse lineage, including fibroblasts (Wu et al., 2006a), renal mesangial cells (McMahon et al., 2002; Mitchell et al., 2004), and epithelial cells (Nascimento-Silva et al., 2007). LXs are protective in several experimental models of disease, e.g., inflammatory bowel diseases (Fiorucci et al., 2004), periodontal disease (Serhan, 2004; Kantarci and Van Dyke, 2005; Kantarci et al., 2006), and cardiovascular disease (Serhan, 2005). LXs have also been reported to act as vasodilators (von der Weid et al., 2004) and may reprogram Mφs from a classically activated (M1) phenotype to a spectrum of alternative activation (Mitchell et al., 2002). The bioactions of LXs are summarized in **Table 1**. The impact of LXs in maintaining the exquisite equilibrium between effective host defense and homeostasis is remarkably illustrated by the fact that over production of LXs may compromise host defense to pathogens. In the case of *Mycobacterium tuberculosis (M.tb),* increased LXA_4_ production is associated with decreased TNF-α activity and results in an inadequate inflammatory response (Tobin et al., 2010). Conversely, LXA_4_ increases survival rate in *Toxoplasma gondii *infection where a compromised immune response due to diminished LO activity and LX biosynthesis is detrimental (Aliberti, 2005).

**Table 1:** Lipoxin induced bioactions.

Cell type	Bioactions* in vitro*
LXA_4_, LXA_4_-analogs and aspirin-triggered lipoxins (ATLs)	
Monocytes	Stimulate chemotaxis and adhesion without causing ROS production (Maddox and Serhan, 1996)
Macrophages	Stimulate efferocytosis while reducing inflammatory cytokine secretion (IFN-γ and IL-6) and increasing pro-resolving cytokine secretion (IL-10) (Mitchell et al., 2002; Schwab et al., 2007)
	Switch Mφ phenotype from inflammatory to pro-resolving
PMN	Inhibit chemotaxis, adhesion, and transmigration (Chiang et al., 2006).
	Inhibit pro-inflammatory cytokine secretion (Jozsef et al., 2002)
	Inhibit ROS production (Levy et al., 1999; Börgeson et al., 2010)
	Enhance CCR5 expression on apoptotic PMN (Ariel et al., 2006)
	Attenuate P-selectin-mediated PMN–endothelial cell interactions (Papayianni et al., 1996)
DCs	Regulated as monocytes differentiate into DCs (Yang et al., 2001)
	Trigger SOCS-2 expression (Machado et al., 2006)
Eosinophils	Inhibit chemotaxis, IL-5, and eotaxin secretion (Soyombo et al., 1994; Bandeira-Melo et al., 2000; Levy et al., 2002)
Platelet	Inhibit *Porphyromonas gingivalis*-induced aggregation (Börgeson et al., 2010)
T cells	Inhibit anti-CD3 Ab induced TNF-α (Ariel et al., 2003)
NK-cells	Block cytotoxicity (Ramstedt et al., 1985, 1987)
PBMC	Inhibit anti-CD3 Ab induced TNF-α (Ariel et al., 2003)
Endothelium	Inhibit P-selectin mobilization (Scalia et al., 1997)
	Upregulate IL-10 while inhibiting LTD_4_ and VEGF stimulated proliferation and angiogenesis (Baker et al., 2009)
Epithelium	Inhibit TNF-α induced IL-8 (Bonnans et al., 2007)
	Inhibit epithelial to mesenchymal transition (Wu et al., 2010)
Fibroblasts	Inhibit proliferation (Wu et al., 2006a)
	Inhibit IL-1β induced IL-6, IL-8, and MMP-3 (Sodin-Semrl et al., 2000)
Mesangial cells	Inhibit inflammatory cytokine production (Wu et al., 2006b), proliferation and cell cycle progression (Badr et al., 1989; Mitchell et al., 2004, 2007; Wu et al., 2005, 2006b) as well as ROS production (Mitchell et al., 2007)
GI epitlelium (enterocytes)	Antagonize TNF-α stimulated neutrophil-enterocyte interactions *in vitro* and attenuate TNF-α chemokine release and colonocyte apoptosis in human intestinal mucosa *ex vivo* (Goh et al., 2001)
	Inhibit TNF-α induced IL-8 (Gewirtz et al., 2002)
Hepatocytes	Reduce PPARα and CINC-1 expression (Planaguma et al., 2002)
Astrocytoma cells	Inhibit IL-1β induced IL-8 and ICAM-1 expression (Decker et al., 2009)
**LXB_4_ and LXB_4_-analogs**	
Monocytes	Stimulate monocytes recruitment, chemotaxis and adherence without causing ROS production (Maddox and Serhan, 1996)
	Increase adherence of undifferentiated THP-1 to laminin (Maddox et al., 1998)
PBMC	Inhibit anti-CD3 Ab induced TNF-α (Ariel et al., 2003)
PMN	Inhibit PMN migration across endothelium (HUVEC monolayer; Maddox et al., 1998)
	Attenuate P-selectin-mediated PMN–endothelial cell interactions (Papayianni et al., 1996)
NK cells	Inhibit cytotoxicity (Ramstedt et al., 1985)

## LIPOXIN RECEPTORS AND SYNTHETIC LIPOXIN ANALOGS

The principal LXA_4_ receptor is FPR2/ALX, which has been identified and cloned in numerous cell types, including monocytes and Mφs (Maddox et al., 1997), T cells (Ariel et al., 2003), synovial fibroblasts (Sodin-Semrl et al., 2000), renal mesangial cells (McMahon et al., 2002), and enterocytes (Gronert et al., 1998). In contrast to conventional GPCRs, which typically show very specific ligand binding, the FPR2/ALX receptor binds pleiotropic ligands, both lipids and small peptides, such as acute phase proteins (Chiang et al., 2000), and may elicit ligand-dependent pro-inflammatory or anti-inflammatory responses (Chiang et al., 2006; Maderna and Godson, 2009). Krishnamoorthy et al. (2010) recently found that LXA_4_ also interacts with another G-protein coupled receptor, namely GPR32.

LXA_4_ undergoes rapid inactivation *in vivo*, primarily by PG dehydrogenase-mediated oxidation and reduction (Serhan et al., 1995) and efforts have been made to design chemically stable LX analogs. Because the three-dimensional molecular structure of the FPR2/ALX receptor is as of yet unknown, designing LXA_4_ analogs is based on experimentally discovered structure/function relationship of LXA_4_. The LXA_4_ molecule can be considered in three regions; the lower chain, the upper chain, and the tetraene side chain (Duffy and Guiry, 2010). The first generation LXA_4_ analogs carry modifications in the lower alkyl chain, to increase metabolic stability and prevent oxidation (Clish et al., 1999). The second generation analogs are collectively referred to as 3-oxa-LXA_4_ and were constructed carrying modifications in the upper chain (Petasis et al., 2005), replacing the C_3_ methylene group with an oxygen molecule (Guilford and Parkinson, 2005). The third generation LXA_4_ analogs are characterized by replacement of the triene structure with a benzene ring (O’Sullivan et al., 2007; Petasis et al., 2008). Importantly, the *o*-[9, 12]-Benzo-15-epi-LXA_4_ has been shown to activate the FPR/ALX receptor in a similar manner to native LXA_4_, using an engineered β-arrestin system (Sun et al., 2009).

## RESOLVINS, PROTECTINS, AND MARSEINS

Resolvins, protectins, and maresins are pro-resolving lipids discovered by Serhan et al. (2000) through sophisticated lipidomic analysis of resolution phase exudates in the murine dorsal air pouch model. Resolvins may be divided into the E series (RvEs) and D series (RvDs), which are generated from eicosapentaenoic acid (EPA) and docosahexaenoic acid (DHA), respectively, the most common forms of ω-3 PUFA. Similarly, protectins and maresins are generated from DHA. Like the LXs, resolvins are generated in a transcellular manner by the sequential action of LO. Protectins and maresins on the other hand are generated by single cells, but also through the action of LO. In neutrophils RvE1 has been shown to bind the GPCR LTB_4_ receptor BLT1 with a K_*d*_ of 45 nM (Arita et al., 2007), whereas in Mφ and dendritic cells RvE1 bind ChemR23 with a K_*d*_ of 11.3 ± 5.4 nM and B_max_ indicating approximately 4,200 binding sites per cell (Arita et al., 2005b; Kohli and Levy, 2009). RvD1 has also been reported to interact both with FPR2/ALX and GPR32 in phagocytes (Krishnamoorthy et al., 2010). As of yet it is not entirely clear which receptor the protectins and maresins act through, although PD1 has a high affinity surface binding site on human PMN and retinal pigment epithelium cells (Bannenberg and Serhan, 2010). Resolvins, protectins, and maresins all display potent anti-inflammatory and pro-resolving effects inhibiting production of pro-inflammatory mediators, regulating neutrophil trafficking and promoting efferocytosis (Schwab et al., 2007; Serhan, 2009). The effects of these lipid mediators are summarized in **Table 2**.

**Table 2:** Resolvin, protectin, and maresin induced bioactions.

Cell type	Bioactions *in vitro*
Resolvin E1	
Macrophages	Stimulates efferocytosis while reducing IFN-γ and IL-6 (Schwab et al., 2007)
PMN	Decreases transendothelial and epithelial migration (Campbell et al., 2007)
	Stimulates L-selectin shedding, while reducing CD18 expression and inhibiting PMN rolling *in vivo* (Dona et al., 2008)
	Attenuates BLT1 depended TNF-α and NF-κB activation (Arita et al., 2007)
	Enhances CCR5 expression on apoptotic PMN (Ariel et al., 2006)
Dendritic cells	Inhibits migration (Arita et al., 2005a)
	Reduces IL-12 production from DCs stimulated with pathogen extract (Arita et al., 2005a)
Platelets	Disruptes platelet aggregation (Dona et al., 2008; Fredman et al., 2010)
**Resolvin D1**	
Microglia cells	Inhibits IL-1β expression (Serhan et al., 2002)
**Protectin D1**	
PMN	Enhances CCR5 expression on apoptotic PMN (Ariel et al., 2006)
Mφ	Stimulates efferocytosis while reducing IFN-γ (Schwab et al., 2007)
T cell	Promotes apoptosis, inhibits TNF-α and IFN-γ (Ariel et al., 2005)
Glia cells	Reduces IL-1β-induced NF-κB activation and COX2 expression (Marcheselli et al., 2003), reduces amyloid β-42-induced nerotoxicity, promotes amyloid β-induced apoptosis (Lukiw et al., 2005)
Epithelium	Protects from apoptosis induced by oxidative stress (Mukherjee et al., 2004)
**Maresin 1**	
Macrophage	Stimulates efferocytosis (Serhan et al., 2009)

## INFLAMMATION AND TYPE 2 DIABETES MELLITUS

Diabetes mellitus (DM) is a serious metabolic disorder of glucose homeostasis reflecting destruction of the β-cells of the pancreas and subsequent lack of insulin production (type 1 DM, T1DM) or decreased target organ sensitivity to insulin and β-cell dysfunction (type 2 DM, T2DM). T2DM is defined as having a fasting plasma glucose ≥7.0 mmol/l and affects over 90% of diabetics, or an estimated 285 million people globally (Cusi, 2010). T2DM imposes significant socioeconomic burdens through its many diabetes-associated complications. These can be divided into microvascular complications [diabetic nephropathy (DN), neuropathy, and retinopathy] and macrovascular complications [atherosclerosis, ischemic heart disease, stroke, and peripheral vascular disease often resulting in amputations] (Wild et al., 2004). Risk factors of T2DM include genetic preposition, ethnicity, high blood pressure, and high cholesterol, but obesity is frequently cited as the primary cause.

The role of inflammation in diabetes is becoming more evident and elevated circulating interleukin (IL)-1β, IL-6, and C-reactive protein (CRP) are predictive of T2DM (Navarro and Mora, 2006; de Luca and Olefsky, 2008; Donath and Shoelson, 2011). These inflammatory markers are primarily derived from the adipose tissue and the liver. The hypothesis that the pathogenesis of T2DM reflects an inflammatory disorder is supported by preclinical studies and clinical trials using anti-inflammatory agents (Donath and Shoelson, 2011). Examples of these include IL-1β receptor blockers, anti-TNF-α and IL-6 therapies, as well as the use of salsalate. We will now briefly discuss current attempts to use anti-inflammatory therapeutics to attenuate the pathology of diabetes.

Interleukin-1β is a key regulator of inflammation both in T1DM and T2DM and has been shown to induce pancreatic β-cell apoptosis and exacerbate the systemic inflammation associated with diabetes, for instance by augmenting adipocyte TNF-α and IL-6 production (Akash et al., 2012). Patients with T2DM display increased IL-1β levels (Boni-Schnetzler et al., 2008), while its naturally occurring IL-1 receptor antagonist (IL-1Ra) is diminished (Maedler et al., 2004). Interest has been directed toward using IL-1Ra as a therapeutic in T2DM. Clinical trials show that the IL-1Ra anakinra improves glycemia and β-cell secretory functions, while attenuating systemic inflammation (Donath and Shoelson, 2011). For instance, anakinra administered over a 13-week period in T2DM patients increased insulin production, while glycosylated hemoglobin, i.e., HbA1c and the inflammatory marker CRP were significantly reduced (Larsen et al., 2007). The limitation with IL-1Ra lies in its short half-life, but successful attempts have been made to increase its stability by fusing IL-1Ra with peptides such as human serum albumin (HLA) or elastin-like polypeptides (ELPs), although these compounds remain to be tested in diabetic models (Akash et al., 2012).

TNF-α is also implicated in the pathogenesis of insulin resistance (IR) and its expression correlates with reduced insulin-stimulated glucose disposal (Kern et al., 2001). TNF-α is elevated both in obese rodents (Uysal et al., 1997) and obese humans (Hotamisligil et al., 1995; Kern et al., 2001) and furthermore decreases upon weight loss (Kern et al., 1995). TNF-α^– / –^ *ob/ob *mice have significantly improved insulin sensitivity (Uysal et al., 1997) and obese mice lacking the TNF-α receptor are protected from high fat diet induced IR (Romanatto et al., 2009). However, in humans TNF-α neutralizing antibodies does not appear to improve insulin sensitivity in obese subjects (Ofei et al., 1996; Rosenvinge et al., 2007). Nevertheless, TNF-α blockers are often used to treat rheumatoid arthritis (RA) and it was recently reported that obese RA patients receiving TNF-α blockers displayed improved fasting glucose and increased circulating adiponectin levels (Stanley et al., 2010), possibly warranting more studies in the field. IL-6 is also an important inflammatory mediator in diabetes and increased levels correlate with IR (Pradhan et al., 2001), although it appears to have a dual role. Whereas IL-6 causes IR in adipocytes (Rotter et al., 2003) and anti-IL-6 therapy over a 6 month period diminished HbA1c in diabetic RA patients (Ogata et al., 2011), the IL-6 derived from skeletal muscle during exercise appears beneficial (Pedersen et al., 2003). The use of anti-IL-6 blockers as an anti-inflammatory therapeutic in diabetes has therefore been debated. Salsalate on the other hand is a very interesting drug in the context of diabetes and has been shown to reduce CRP, FFA, and triglycerides while increasing insulin sensitivity and adiponectin levels (Koska et al., 2009; Goldfine et al., 2010). Salsalate may, however, cause gastric irritation and should be used with caution in pregnancy (Torloni et al., 2006; Chyka et al., 2007). Collectively these studies indicate the potential of using anti-inflammatory therapeutics to attenuate T2DM.

## RESOLUTION OF ADIPOSE INFLAMMATION IN TYPE 2 DIABETES MELLITUS

There is a growing appreciation that adipose tissue is not merely an insulating energy store but is actually an endocrine organ regulating appetite, glucose and lipid metabolism, blood pressure, inflammation, and immune function (Kershaw and Flier, 2004). Adipose tissue has been shown to play a particularly important role in the systemic inflammation associated with obesity, IR, and diabetes. Factors such as prolonged obesity or aging cause a state of systemic low-grade inflammation, which induces monocyte recruitment to the adipose tissue. Adipose tissue is a source of pro-inflammatory cytokines and adipose tissue Mφ (ATM) derived TNF-α, IL6, and IL-1β contribute to adipose IR and exacerbates systemic inflammation (Lumeng et al., 2007b). Promoting resolution of adipose inflammation would likely be a beneficial therapeutic approach, reducing the risk of developing obesity-associated complications, such as IR and T2DM (Donath and Shoelson, 2011).

Given the spectrum of anti-inflammatory and pro-resolution bioactions of LXs and other counter-regulatory lipid mediators, these may provide a potential intervention to attenuate adipose inflammation (Gonzalez-Periz and Claria, 2010). We recently reported a role of LXA_4_ in adipose inflammation, culturing adipose explants of aging mice as an *ex vivo* model of adipose inflammation (Börgeson et al., 2012). We confirmed that LXA_4_ increased expression of critical components of insulin sensitivity, including the glucose transporter GLUT-4 and IRS-1, consistent with restoring insulin sensitivity in the tissue. Furthermore, LXA_4_ decreased IL-6 secretion while increasing production of the pro-resolving IL-10, suggesting that LXA_4_ acted in a pro-resolving manner (Börgeson et al., 2012). Indeed, IL-10 inversely correlates with T2DM and has been shown to inhibit IL-6-induced IR, attenuate MCP-1 secretion, and promote GLUT-4 and IRS-1 expression (Lumeng et al., 2007a; Gonzalez-Periz and Claria, 2010). The study also demonstrated that LXA_4_ partially rescued MΦ-inhibited adipose glucose uptake *in vitro* (Börgeson et al., 2012). Inflammatory MΦs are a key component of augmented adipose IR (Lumeng et al., 2007b; Cusi, 2010; Spencer et al., 2010). Importantly, LXA_4_-mediated reversal of insulin desensitization correlated with restored adipose Akt activation, which is necessary for translocation of the glucose sensitizing GLUT-4 receptor from the cytosol to the plasma membrane (Börgeson et al., 2012). Interestingly, RvD1 also increased insulin-stimulated pAkt in adipose tissue of obese *db/db* mice (Hellmann et al., 2011). Furthermore, LXA_4_ inhibited MΦ TNF-α production, which is an important cytokine previously demonstrated to inhibit adipose glucose uptake *in vitro* (Gao et al., 2003). LXA_4_ also inhibited MCP-1 secretion, though the importance of MCP-1 in adipose inflammation has been debated (Chen et al., 2005; Inouye et al., 2007). The reduction of inflammatory cytokines may suggest that LXA_4_ promoted restoration of insulin sensitivity by altering MΦ phenotype toward resolution. Finally, LXA_4_ also appeared to have a direct impact on adipocytes as it rescued TNF-α-induced desensitization to insulin-stimulated Akt activation, which also correlated with increased GLUT-4 translocation.

The beneficial effects of ω-3 PUFA, RvE1, and PD1 have also been shown in *ob/ob* mice (Gonzalez-Periz et al., 2009). Both ω-3 PUFA enriched diet and intraperitoneal injections of RvE1 increased expression of genes involved in glucose transport (GLUT-4) and insulin signaling (IRS-1), as well as genes involved in insulin sensitivity (PPARγ). Similar to ω-3 PUFA, RvE1 increased adiponectin levels, as did PD1 when incubated with adipose explants from *ob/ob* mice (Gonzalez-Periz et al., 2009). Additional studies show that RvD1 decrease accumulation of ATMs and improve insulin sensitivity while reducing fasting blood glucose in *db/db* diabetic mice (Hellmann et al., 2011). Interestingly, the total number of ATMs remained unaltered with RvD1 treatment, but the ratio of M2:M1 increased. The number of adipose crown like structures (CLS) in obese animals was also reduced by 50–60% (Hellmann et al., 2011) and RvD1 significantly increased circulating adiponectin and adipose phosphorylation of AMPK. The study also reports diminished IL-6 secretion (Hellmann et al., 2011), which has previously been shown to suppress adiponectin in 3T3-L1 adipocytes (Fasshauer et al., 2003) and may explain the restored adiponectin levels, which in turn have been shown to increase insulin sensitivity (Kristiansen and Mandrup-Poulsen, 2005; Kadowaki et al., 2006).

## INFLAMMATION AND DIABETIC NEPHROPATHY

Diabetic nephropathy presents a particularly important problem as it develops in 25–40% of diabetic patients and is the major cause of end-stage kidney disease (Ritz et al., 1999). DN is a type of chronic kidney disease (CKD) rising in prevalence in concert with chronic DM in susceptible individuals. In addition to being the leading cause of renal failure, T2DM is also an independent risk factor in the development of cardiovascular disease (Syed and Khan, 2011). DN reflects the convergence of inflammatory, metabolic, and hemodynamic factors. Inflammation causes glomerulosclerosis, tubular atrophy, damage to renal vasculature, and fibrosis (Ferenbach et al., 2007). Renal matrix accumulation arises in response to paracrine and autocrine mediators produced by resident and infiltrating cells, such as mesangial cells and Mφs.

Promoting inflammatory resolution is likely an attractive approach when trying to prevent renal fibrosis and CKD (Börgeson and Godson, 2010). The mechanisms by which resolution of renal inflammation occurs naturally and how they are subverted in disease are only beginning to be understood. Important components include efferocytosis of apoptotic cells and a change of the cytokine milieu from pro-inflammatory to anti-inflammatory/pro-resolving (Ferenbach et al., 2007; Börgeson and Godson, 2010). Biphasic regulation of renal inflammation and NF-κB also appears important, where the first peak mediated through p65/p50 heterodimers induces inflammation through pro-inflammatory mediators such as MCP-1 and RANTES. The second peak on the other hand (p50/p50 homodimers) promotes resolution by downregulating MCP-1/CCL1, RANTES/CCL5, and TNF-α (Panzer et al., 2009), while inducing expression of pro-resolving IL-10 (Cao et al., 2006). Similarly, to other pathologies it also appears that the phenotype of Mφs is important in CKD (Wada et al., 2004; Sung et al., 2007). Whereas M1 Mφ are detrimental, the M2a and perhaps even more so the M2c phenotype is beneficial (Wang and Harris, 2011).

Mφs play an important role in DN as previously reported by Tesch (2008), 2010. Mφs increase production of ROS, pro-inflammatory cytokines, and pro-fibrotic growth factors that contribute to the formation of myo-fibroblasts. Mφs also appear to directly activate fibroblasts to a pro-fibrotic (myo-fibroblast) phenotype through secretion of galectin-3 (Henderson et al., 2008). Inhibition of Mφ recruitment has been suggested to attenuate disease in several models of renal fibrosis with varying efficacy (Wada et al., 2004; Sung et al., 2007). For instance, MCP-1^– / –^ mice are protected against renal injury in a model of T1DM (Chow et al., 2006) and furthermore urinary levels of MCP-1 are predictive of renal injury in humans and have been proposed as a diagnostic marker of progressive diabetic kidney disease (Tesch, 2008). There is a growing appreciation that the plasticity of Mφs is an important factor in disease progression (Duffield, 2011; Wang and Harris, 2011) and that Mφs also contribute to the resolution of renal inflammation. For instance, Mφ efferocytosis of apoptotic cells is coupled to the generation of anti-inflammatory mediators such as IL-10 (Ricardo et al., 2008). To this effect, re-programming Mφs *ex vivo* toward a M2 phenotype (IL-4/IL-13 stimulation) provides protection in models of renal disease, whereas the M1 phenotype (LPS stimulation) is detrimental (Wang et al., 2007). Additional research suggests that M2a and M2c phenotypes are both renoprotective, but that the latter appears to be the more effective (Wang and Harris, 2011).

## EXPERIMENTAL THERAPEUTICS AND DN

Diabetic nephropathy is a chronic disease and current therapeutics primarily focus on glycemic and blood-pressure control through drugs targeting the renin–angiotensin system (RAS), such as angiotensin-converting-enzyme (ACE) inhibitors and angiotensin receptor blockers (ARBs). However, these treatment regimes only slow the progression of the disease, but do not halt or reverse it. Furthermore, prolonged use of RAS inhibitors may induce hyperkalemia, reduction in systemic blood pressure and decreased renal blood flow. Therefore, there is a profound need for novel therapeutic strategies in this field and the search is ongoing (Decleves and Sharma, 2010; Shepler et al., 2012). Examples of experimental therapeutics that show potential include bardoxolone methyl, which in clinical trials increases estimated glomerular filtration rate (eGFR) and creatinine clearance, while inhibiting inflammation in diabetic patients with stage 3b-4 CKD (Pergola et al., 2011a,b; Thomas and Cooper, 2011). Pirfenidone is an oral anti-fibrotic and anti-inflammatory agent which shows therapeutic potential in DN, although it was initially developed for treatment of idiopathic pulmonary fibrosis. In a randomized, double blind study pirfenidone increased eGFR and decreased markers of inflammation (TNF, INF-γ, and IL-1; Sharma et al., 2011) and has also demonstrated anti-fibrotic potential in both *in vitro* (Hewitson et al., 2001) and *in vivo* (RamachandraRao et al., 2009; Takakuta et al., 2010) models of renal disease. Vitamin D analogs, e.g., paricalcitol, may also be renoprotective agents through negatively regulating the RAS system and attenuation of renal fibrosis in rodent unilateral ureteric obstruction (UUO) models inhibiting accumulation of ECM as well as TGF-β1 and MCP-1 gene expression signaling (Li and Batuman, 2009; Li, 2010). Vitamin D analogs have also been suggested to prevent podocyte injury by promoting expression of slit diaphragm proteins (Li, 2011) and shows promising potential in emerging clinical trials reducing proteinuria in CKD patients (Li, 2010).

As inflammation is a common denominator in CKD and a hallmark of DN, pro-resolving therapeutics may have potential benefit. We recently reported that LXs are protective in CKD, as pre-treatment with LXA_4_ and benzo-LXA_4_ modulates inflammation and fibrosis in early UUO-induced injury (Börgeson et al., 2011a). UUO is an established model of progressive tubulointerstitial fibrosis and inflammation, relevant to CKD of diverse etiologies, including DN. UUO induces marked Mφ infiltration, tubular cell death, fibroblast activation, and possible phenotypic transition of resident renal cells characteristic of progressive renal fibrosis (Higgins et al., 2007; Chevalier et al., 2009). Benzo-LXA_4_ and LXA_4_ attenuated UUO-induced fibrotic responses such as collagen accumulation by inhibiting collagen-1α2 gene expression, expression of collagen chaperone HSP47 and TGF-β1 signaling pathways (Börgeson et al., 2011a). Interestingly, RvD1 has also been demonstrated to attenuate collagen deposition in a murine model of renal ischemia reperfusion (Duffield et al., 2006). Specifically, LXs inhibited UUO-induced TGF-β1 canonical (Smad2) and non-canonical (Akt, Erk, and p38MAPK) signaling pathways, translating to reduced pro-fibrotic signaling (Börgeson et al., 2011a). Although LXA_4_ did not alter the expression of TGF-β1, it did inhibit expression of MMP2 and CTGF. This is indeed noteworthy since MMP2 activates latent TGF-β1 and is a major driver of TGF-β1-mediated fibrosis. The LXA_4_ mediated reduction of CTGF, both at mRNA and protein levels, would likely result in reduced fibrotic responses. The anti-fibrotic effect of LXs has been demonstrated in several *in vitro* systems, inhibiting proliferation and cell cycle progression in mesangial cells (Börgeson and Godson, 2010). Recent data also demonstrate protection by RvE and RvD in murine UUO (Qu et al., 2012). LXs also appeared to shift Mφ phenotype and displayed significant pro-resolving actions in UUO-induced CKD. Whereas the total number of Mφ and MCP-1 remained unaltered, LX treated animals displayed decreased pro-inflammatory IFN-γ and TNF-α cytokines and increased pro-resolving IL-10 levels (Börgeson et al., 2011a). Indeed, it appeared that the LXs induced a shift the Mφ phenotype toward an early stage M2c reparative phenotype, based on the high IL-10 expression induced by benzo-LXA_4_, although TGF-β1 remained unaffected (Börgeson et al., 2011a).

Micro RNA (miRNA) may also prove an important therapeutic target in DN, as they have demonstrated importance in CKD pathogenesis (Kato et al., 2007; Wang et al., 2008; Long et al., 2010). We recently reported that whereas TGF-β1 downregulates expression of the miRNA let-7c in renal epithelia, LXA_4_ enhances let-7c expression, and attenuates TGF-β1 fibrotic responses as let-7c targets expression of the TGF-βR1 (Brennan et al., in revision). Importantly, LXs inhibit ROS production (Börgeson and Godson, 2010; Börgeson et al., 2011b; Wu et al., 2012), which may be analogous to the antioxidant effect of bardoxolone methyl (Rojas-Rivera et al., 2012). Indeed, bardoxolone methyl is an antioxidant inflammation modulator (AIM) compound, targeting Nrf2 which is a master regulator of the antioxidant response. Interestingly, LXA_4_ has been shown to inhibit LPS-mediated ROS production and to downregulate Nrf2 protein levels in human umbilical vein endothelial cells (HUVECs; Pang et al., 2011).

## CONCLUSION

Increasing evidence supports the role of chronic inflammation in the pathogenesis of T2DM and associated complications such as DN. Pro-resolving mediators, such as LXs, resolvins, and protectins, attenuate diabetes-related pathologies, including kidney disease and adipose inflammation. Thus promoting the resolution of inflammation through use of these lipids may provide a novel therapeutic strategy in the fight against diabetes-related pathologies.

## Conflict of Interest Statement

The authors declare that the research was conducted in the absence of any commercial or financial relationships that could be construed as a potential conflict of interest.

## Abbreviations

ACE inhibitors: angiotensin-converting-enzyme inhibitors
AIM: antioxidant inflammation modulator
ARBs: angiotensin receptor blockers
ATMs: adipose tissue Mφs
CKD: chronic kidney disease
CLS: crown like structures
CRP: C-reactive protein
DHA: docosahexaenoic acid
DN: diabetic nephropathy
eGFR: estimated glomerular filtration rate
EPA: eicosapentaenoic acid
HUVECs: human umbilical vein endothelial cells
IL: interleukin
LO: lipoxygenase
LXA_4_: lipoxin A_4_ (S),6(R),15,Trihydroxyeicosa-7E,9E,11Z,13E-tetraenoic acid
M.tb: *Mycobacterium tuberculosis*
maresins: MΦ mediators in resolving inflammation
miRNA: micro RNA
Mφ: macrophages
PG: prostaglandin
PMN: polymorphonuclear cell
RA: rheumatoid arthritis
RAS: renin–angiotensin system
Rvs: resolvins
T2DM: type 2 diabetes mellitus
UUO: unilateral ureteric obstruction.
